# Both Free Indole-3-Acetic Acid and Photosynthetic Performance are Important Players in the Response of *Medicago truncatula* to Urea and Ammonium Nutrition Under Axenic Conditions

**DOI:** 10.3389/fpls.2016.00140

**Published:** 2016-02-16

**Authors:** Raquel Esteban, Beatriz Royo, Estibaliz Urarte, Ángel M. Zamarreño, José M. Garcia-Mina, Jose F. Moran

**Affiliations:** ^1^Laboratory of Plant Physiology and Agrobiology, Institute of Agrobiotechnology, IdAB-CSIC-UPNA-Government of Navarre, Public University of NavarreMutilva, Spain; ^2^Group—CMI Roullier, Department of Environmental Biology, Agricultural Chemistry and Biology, University of NavarrePamplona, Spain

**Keywords:** ammonium, auxin, OJIP curve, nitrate, root system architecture, urea

## Abstract

We aimed to identify the early stress response and plant performance of *Medicago truncatula* growing in axenic medium with ammonium or urea as the sole source of nitrogen, with respect to nitrate-based nutrition. Biomass measurements, auxin content analyses, root system architecture (RSA) response analyses, and physiological parameters were determined. Both ammonium and ureic nutrition severely affected the RSA, resulting in changes in the main elongation rate, lateral root development, and insert position from the root base. The auxin content decreased in both urea- and ammonium-treated roots; however, only the ammonium-treated plants were affected at the shoot level. The analysis of chlorophyll *a* fluorescence transients showed that ammonium affected photosystem II, but urea did not impair photosynthetic activity. Superoxide dismutase isoenzymes in the plastids were moderately affected by urea and ammonium in the roots. Overall, our results showed that low N doses from different sources had no remarkable effects on *M. truncatula*, with the exception of the differential phenotypic root response. High doses of both ammonium and urea caused great changes in plant length, auxin contents and physiological measurements. Interesting correlations were found between the shoot auxin pool and both plant length and the “performance index” parameter, which is obtained from measurements of the kinetics of chlorophyll *a* fluorescence. Taken together, these data demonstrate that both the indole-3-acetic acid pool and performance index are important components of the response of *M. truncatula* under ammonium or urea as the sole N source.

## Introduction

Nitrate (NO${}_{3}^{-}$), ammonium (NH${}_{4}^{+}$), and urea [CO(NH_2_)_2_] are the main forms of nitrogen (N) present in agronomic fertilizers and, thus, are the principal sources of N taken up by crops. However, in agronomic soils, the scenario is much more complex because plants are not the only consumers of these nutrients, which can also be taken up and transformed by microorganisms in the rhizosphere. For instance, urea can be rapidly degraded by the soil microbiota in a manner that depends on the availability of microbial species and environmental conditions, such as temperature (Bremmer and Krogmeier, 1988). Additionally, the presence of nitrifying microorganisms, such as *Nitrosomonas* and *Nitrobacter* species, can lead to differential production of NO${}_{3}^{-}$ (Hollocher, 1981; Ferguson et al., 2007). This NO${}_{3}^{-}$ production often contributes positively to plants, as low NO${}_{3}^{-}$ contents in ammonium-containing media can trigger positive signaling effects on plants and, hence, reduce NH${}_{4}^{+}$ stress (Houdusse et al., 2005; Garnica et al., 2010; Hachiya et al., 2012). Moreover, soil microorganisms have been demonstrated to act as hormone producers, especially for auxins, gibberellins, cytokinins, and abscisic acid (Reddy and Saravanan, 2013). All of these facts increase complexity when studying N nutrition in agronomic models. Thus, growing plants and modeling their response in axenic cultures is of great importance for assessing the ability of plants to use and respond to different N sources.

Once NO${}_{3}^{-}$ is assimilated, it must be reduced to NH${}_{4}^{+}$ before it can be incorporated into amino acids, with consequent redox/energy consumption, while NH${}_{4}^{+}$ can directly be assimilated, with a lower energy cost. Therefore, for the plant economy, NH${}_{4}^{+}$ may be thought of as a favorable N nutrient. However, the presence of NH${}_{4}^{+}$ as the only N source is paradoxically toxic to most plants, and plant biomass, phenotypic changes and the plant stress response depend on the concentration and the relative tolerance capacity of the plant species or variety (Gerendás et al., 1998; Cruz et al., 2011; Li et al., 2014). Although the mechanisms of toxicity are not fully resolved, futile cycling associated with passive diffusion of NH${}_{4}^{+}$ across the plasma membrane, followed by NH${}_{4}^{+}$ extrusion, appeared to be responsible for an unfavorable energetic balance in plant cells (Britto and Kronzucker, 2002). Subsequent data on the uptake of ^15^N-labeled NH${}_{4}^{+}$ and the natural abundance of the isotope in plants revealed passive transport of ammonia gas through plant cell membranes, which is correlated with the susceptibility of plants to ammonia (Ariz et al., 2011b). This evidence was further confirmed through flux analyses with the short-lived radioisotope ^13^N (Coskun et al., 2013). Ammonium nutrition has also been reported to modify the redox state in several species, consequently changing the homeostasis of reactive oxygen species (ROS; Medici et al., 2004; Patterson et al., 2010). Increases in mitochondrial ROS levels in leaves have been described as being associated with NH${}_{4}^{+}$ stress in some plants, such as *Arabidopsis* (Podgórska et al., 2013; Podgórska and Szal, 2015). However, it was concluded that the ammonium-generated stress observed in spinach and pea plants was not oxidative stress (Domínguez-Valdivia et al., 2008). Hence, the relationship of NH${}_{4}^{+}$ with oxidative stress in plants is still an open question (Bittsánszky et al., 2015).

The possible toxicity of urea is also a matter of debate. In general, similar effects on plants to those described for NH${}_{4}^{+}$ have been reported for urea, but with a lower intensity (Houdusse et al., 2005). Although the understanding of NH${}_{4}^{+}$ utilization has improved in the last two decades (Gerendás et al., 1998; Witte et al., 2002; Mérigout et al., 2008; Zanin et al., 2014, 2015), the physiological aspects of urea acquisition in plants grown under axenic conditions have only been investigated in *Arabidopsis* and rice (Wang et al., 2013; Yang et al., 2015). Therefore, there are still important gaps in our knowledge of the mechanisms of nitrogen nutrition in plants under axenic conditions, including the role of urea as an accessible nitrogen source for crops.

Here, we report a standardized method for growing the model legume *Medicago truncatula* in axenic culture using NO${}_{3}^{-}$, NH${}_{4}^{+}$, or urea as the sole source of nitrogen, managing control of the pH, and growing conditions. The physiological characteristics of this species in association with urea nutrition are unknown. Moreover, this species, which belongs to the family Leguminosae, has been described as quite tolerant to exclusive NH${}_{4}^{+}$ nutrition, although legumes also suffer from phenotypic and physiological changes under different N sources (Domínguez-Valdivia et al., 2008; Ariz et al., 2011a). This tolerance could be related to (i) the ability to fix nitrogen through endosymbiosis with soil bacteria forming nodules and to (ii) the capacity to assimilate the ammonium produced by the endosymbiont. During the growth of legumes with N fixed as the only N form, the endosymbiont may produce oxidative forms of N that may have effects during the growth of the plants. The present study, performed in axenic cultures, allowed the exclusion of contaminating bacteria, thus avoiding nodulation, and the procedures for the analysis of comparative N nutrition were standardized. Under these conditions, we observed a differential phenotypic response to the N source, with a portion of the response being associated with photosynthetic performance and the auxin contents of the plants.

## Materials and methods

### The plant growth system

**Figure 1:**
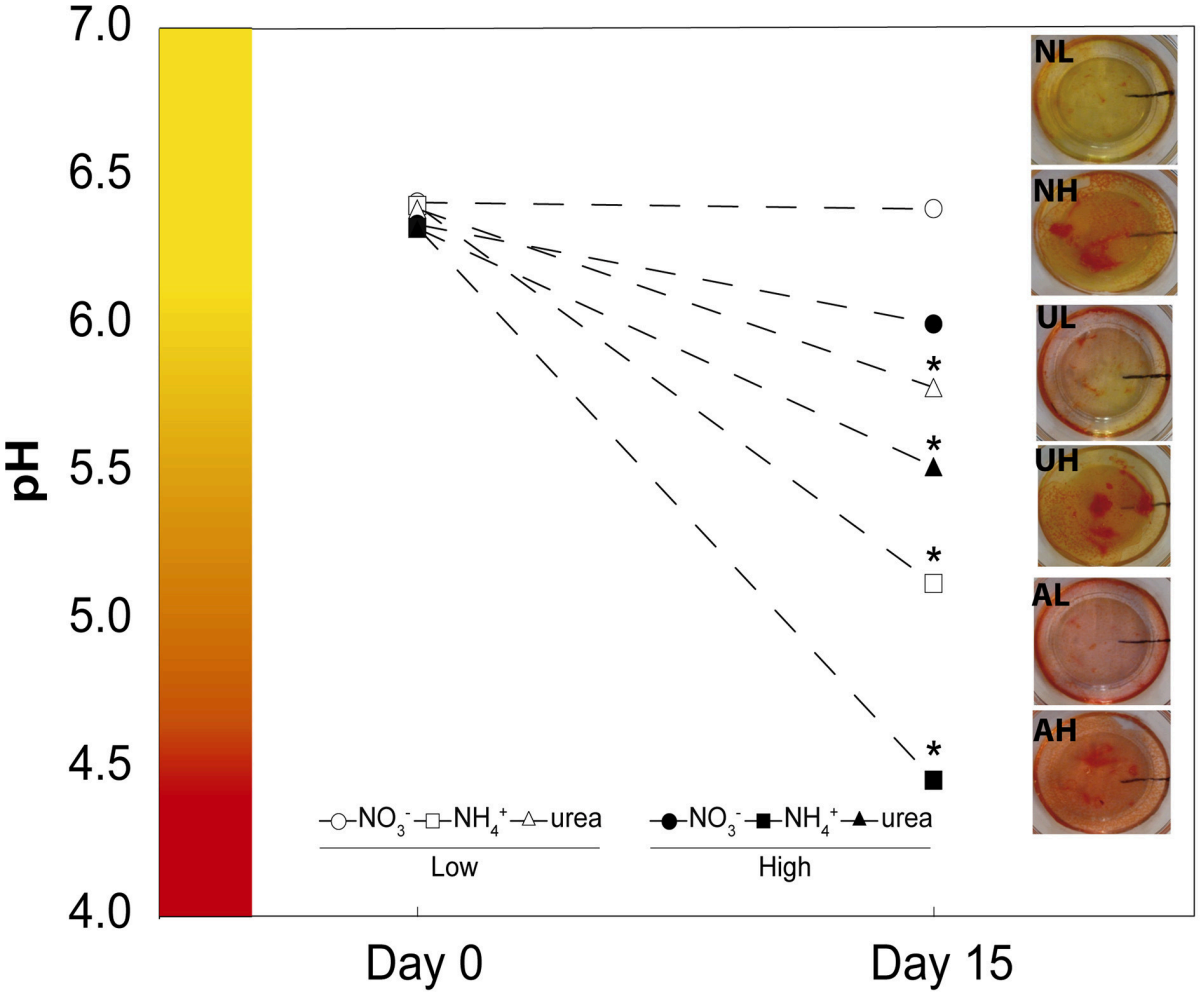
**Effects of NO${}_{3}^{-}$, NH${}_{4}^{+}$, and urea on pH**. pH on days 0 and 15 of the experiment under the different treatments: low doses of NO${}_{3}^{-}$, NH${}_{4}^{+}$, and urea (closed circle, square, and triangle, respectively) and high doses of NO${}_{3}^{-}$, NH${}_{4}^{+}$, and urea (opened circle, square, and triangle, respectively). The values are the mean ± S.E (*n* = 15). Photographs of the pH indicator methyl red on day 15 of the experiment for each of the treatments are shown (NL, AL, UL: low doses of NO${}_{3}^{-}$, NH${}_{4}^{+}$, and urea, respectively, and HH, AH, UH: high doses of NO${}_{3}^{-}$, NH${}_{4}^{+}$, and urea, respectively).

Seeds of *M. truncatula* Gaertn. ecotype *Jemalong* were scarified with 95% sulfuric acid for 8 min and then washed with sterile water and further surface sterilized with 50% sodium hypochlorite for 5 min, followed by washing again with sterile water until the pH reached 7. The seeds were subsequently germinated on 0.4% agar (w/v) plates at 14°C in darkness for 72 h. The germinated seeds were transferred in a sterile laminar flow cabinet to glass pots (five per pot) containing 100 ml of Fahraeus medium with 5 g l^−1^ of phytagel as a nutrient medium. This medium contained 0.9 mM CaCl_2_, 0.5 mM MgSO_4_, 0.7 mM KH_2_PO_4_, and 0.8 mM Na_2_HPO_4_, as macronutrients and 20 μM ferric citrate, 0.8 μM MnCl_2_, 0.6 μM CuSO_4,_0.7 μM ZnCl_2_, 1.6 μM H_3_BO_3_, and 0.5 μM Na_2_MoO_4_, as micronutrients. Nitrogen was applied as NO${}_{3}^{-}$ using Ca (NO_3_)_2_, or as NH${}_{4}^{+}$ using (NH_4_)_2_SO_4_ or as urea. The ammonia- and urea-fed plants were supplemented with 0.5 or 12.5 mM CaSO_4_ to compensate for the Ca^2+^ supplied together with the NO${}_{3}^{-}$ treatment. Two different scenarios were explicitly considered: a low N supply (1 mM) and a high N concentration (25 mM). The lowest N-dose was selected following the previous results of the group, in order to have a non-limiting N availability (Ariz et al., 2010). The highest dose was increased to 25 mM, as in agar media higher doses are usually necessary to have an excess of NH${}_{4}^{+}$ (Li et al., 2010). During the preparation of the axenic media, the pH was controlled in all cases before and after autoclaving because we have observed that autoclaving some solutions differentially affects the pH of the media (data not shown). Thus, the CaCl_2_ and N sources were sterilized by filtering through cellulose acetate filters and added to the rest of medium after autoclaving. This process allowed us to maintain a specific pH = 6.5 at the time of the sowing of seeds (Figure 1). The plants were grown in a growth chamber for 15 days at a day/night temperature of 24.5/22°C, under 80% relative humidity, with a 16/8 h day/night photoperiod and 70 μmoles m^−2^ s^−1^ of photosynthetically active radiation. The pH was controlled before the experiment and after the growth of the plants, and it was adjusted if necessary at the beginning of the experiment. Phosphate buffer was chosen in order to avoid the use of N-based pH buffers. An aliquot of each medium was collected under sterile conditions after autoclaving and the pH was measured (pH day 0) using a Basic 20 pH-meter (Crison). After the harvesting of the plant material, the pH was also measured (pH day 15) directly in the pots. Five replicates of each treatment and time were measured to obtain the results shown in Figure 1. After each harvest, the agar media in all pots was routinely stained with 5 ml of a 0.1% (w/v) solution of methyl red as a pH indicator (Figure 1). When any pot exhibited pH levels outside the range shown in Figure 1 (outside the range of the average ± S.E) was excluded. The sterility monitoring over the 15 days was performed by carefully controlling the agar medium, when no microorganism grown was appreciated the medium was used for harvesting and further analysis. Harvesting was always conducted at 6 h after the onset of the light period. A pool of five plants from different pots was collected (shoots and roots), weighed, and oven dried or frozen in liquid N, and stored at −80°C for further analyses.

### Growth determination: Total biomass, dry weight, plant length, and root system architecture

During harvesting, 15 plants from each treatment were randomly selected to determine the fresh weight of both the shoots and roots. The dry weight was determined after incubation in a dry oven at 80° C for 2 days. The shoot length was measured in these plants as the distance from the shoot base to the apical meristem. The quantification of root growth and architecture was performed with the semi-automated image analysis software SmartRoot (Lobet et al., 2011) by estimating growth rates, the accumulated length of lateral roots and the position of insertion from the root base. For this, five *M. truncatula* plants were grown as previously described on agar plates, and their root system was photographed every 4 days during 15 days (2D photographs). A dataset containing architectural descriptions of the root system under the different N sources was established.

### Auxin content

The concentration of indole-3-acetic acid (IAA) was analyzed in shoot and root extracts using high performance liquid chromatography-electrospray-mass spectrometry (HPLC-ESI-MS/MS) as described in Jauregui et al. (2015). The extraction and purification of this hormone was carried out using the following method also described in Jauregui et al. (2015). Hormones were quantified by HPLC-ESI-MS/MS using an HPLC (2795 Alliance HT; Waters Co., Milford, MA, USA) coupled to a 3200 Q-TRAP LC/MS/MS System (Applied Biosystems/MDS Sciex, Ontario, Canada), equipped with an electrospray interface. A reverse-phase column (Synergy 4 mm Hydro-RP 80A, 150 × 2 mm; Phenomenex) was used. The detection and quantification of the hormone was carried out using multiple reactions monitoring in the negative-ion mode, employing multilevel calibration curves with the internal standards as described in Jauregui et al. (2015).

### Chlorophyll *a* fluorescence rise kinetics measurements

Measurements of chlorophyll *a* fast fluorescence transients (OJIP) were performed in *M. truncatula* leaves by a FluorPen FP 100 (Photon System Instrument, Brno, Czech Republic). This technique allows an estimation of photosynthetic performance and denotes the flow of energy through photosystem II, which is a highly sensitive signature of photosynthesis (Strasser et al., 2000). For a more detailed review, see Strasser et al. (2000, 2004) and Stirbet and Govindjee (2011). Prior to measurements, leaves were dark-adapted for a night period (14 h), to allow the complete relaxation or oxidation of reaction centers in order to determine *F*o. For excitation, bandpass filters of light emitting diodes with 697–750 nm provided of 3000 μmol photons m^−2^ s^−1^ at leaf sample and fluorescence transients were induced and recorded during 2 s at a frequency of 10 μs, 100 μs, 1 ms, and 10 ms for the time intervals of 10–600 μs, 0.6–14 ms, 14–100 ms, and 0.1–2 s, respectively. The fluorescence values at 40 μs (F_*o*_, step 0, all reaction centers of the photosystem II are open), 100 μs (F_100_), 300 μs (F_300_), 2 ms (step J), 30 ms (step I), and maximal (F_*M*_, step P, closure of all reaction centers) were taken into consideration. Cardinal points of the OJIP curve and derived parameters were calculated with the Fluorpen 2.0 software, based on the theory of energy fluxes in biomembranes by the formulas derived from Strasser et al. (2000, 2004). In this paper, we have considered fluorescence parameters derived from the extracted data and (i) normalized signals as V_j_ (F_j_–F_o_/F_M_–F_o_) and V_i_ (F_i_–F_o_/F_M_–F_o_), (ii) quantum yields and efficiencies, (iii) the specific fluxes per active reaction center (RC) for absorption (ABS/RC), trapping (TR_0_/RC), electron transport (ET_0_/RC), and dissipation (DI_0_/RC). We have also analyzed the performance index, Pi_Abs_, which is the potential performance index for energy conservation from photons absorbed by photosystem II to the reduction of intersystem electron acceptors. In this paper, we do not analyze the events relative to PSI (Zubek et al., 2009). The formulas used to calculate the above parameters and detailed information are given in the Supplementary Table S1.

### Superoxide dismutase activity (SOD)

*In gel* SOD activity of roots and shoots of *M. truncatula* was measured as described by Beauchamp and Fridovich (1971), and Asensio et al. (2011, 2012). SOD isoforms identification were made according to known mobility of SOD on native gels and based on the differential inhibition of SOD activity on gels pre-incubated with either 3 mM KCN, which inhibited the CuZnSODs, or 5 mM H_2_O_2_ for 1 h, which inhibited FeSOD (Asensio et al., 2011, 2012). The *in-gel* SOD activity assays were performed at least three times in order to ensure the consistency of the results.

### Metabolite analyses

The reduced and oxidized forms of the antioxidant metabolites (ascorbate, glutathione, and homoglutathione) were determined in shoots and roots as described in Zabalza et al. (2007). In brief, frozen samples (0.2 g) were ground in a mortar with liquid nitrogen and then homogenized with 2 ml extraction buffer (2% metaphosphoric acid; 1 mM EDTA). The homogenate was centrifuged at 4400 g for 2 min at 4°C and filtered. Ascorbate (ASC), glutathione (GSH), and homoglutathione (hGSH) contents were analyzed by high-performance capillary electrophoresis in a Beckman Coulter P/ACE system 5500 (Fullerton, CA) using capillary tubing (50 mm; 30/37 cm long). ASC, GSH, and hGSH were detected with a diode array detector by setting wavelength at 265 for ASC, and 200 nm for reduced thiol tripeptides. To determine dehydroascorbate (DHA), oxidized glutathione (GSSG), and oxidized hGSH (hGSSG) the extracts were reduced with DTT as described by Davey et al. (2003), then total ASC, GSH, and hGSH were analyzed by HPCE using standards as reference. DHA, GSSG, and hGSSG contents were determined as the difference between the total antioxidant pools and levels of their reduced forms. Redox status was determined as the ratio between the reduced form (ASC, GSH, or hGSH) and the total antioxidant content (oxidized + reduced). In this paper, we have considered the pool of GSH and hGSH together due to they are homologous molecules regarding the plant redox state. Protein contents were quantified using a dye-binding Bradford micro-assay (Bio-Rad, Watford, UK) with bovine serum albumin as a standard. At harvest, the relative content of chlorophyll in leaves (SPAD index) was determined using the chlorophyll meter SPAD-502 (Minolta, Japan). This instrument provides a relative measurement of chlorophyll in leaves through the evaluation of the changes of the transmittance in the 600–700 nm region of the visible spectra and in the near infrared region of the spectra.

### Statistics

Differences among treatments were evaluated with one-way ANOVA and *post-hoc* Student-Newman-Keuls test. All data were tested for normality (Kolmogorov–Smirnof test) and homogeneity of variances (Cochran test) and log-transformed if necessary. When this failed to meet ANOVA assumptions, they were analyzed using the nonparametric Mann–Whitney test. The resulting *p*-values were considered to be statistically significant at α = 0.05. Linear regressions were used to analyze relationship in **Figure 7**. Calculated *p*-values, coefficients and regression lines are indicated whenever significant at α = 0.05. Statistical analyses were performed with IBM SPSS Statistics for Windows, Version 21.0. Armonk, NY: IBM Corp.

## Results

### The plant growth system

Previous experiments performed by our group revealed that Murashige and Skoog medium is incompatible with agar solidification under a high NH${}_{4}^{+}$ concentration (data not shown). Therefore, an original differentially N-supplemented axenic medium was used to grow *M. truncatula* plants under axenic conditions with NO${}_{3}^{-}$, NH${}_{4}^{+}$, or urea for 15 days. The growth medium, containing 1.5 mM phosphate buffer, exhibited a significant buffering capacity, similar to that of Murashige and Skoog medium, which contains 1.25 mM phosphate. Hence, the pH of the growth medium during the experiment changed little for the plants grown using NO${}_{3}^{-}$. However, in the plants grown with NH${}_{4}^{+}$ as the sole source of N, there was a significant decrease in pH, with pH measurements of 5.2 and 4.5 being observed for the 1 mM and 25 mM ammonium treatments, respectively. Intermediate decreases were found in association with urea nutrition at both doses (Figure 1).

### Differential root system architecture and growth of seedlings under the tested N sources

**Figure 2:**
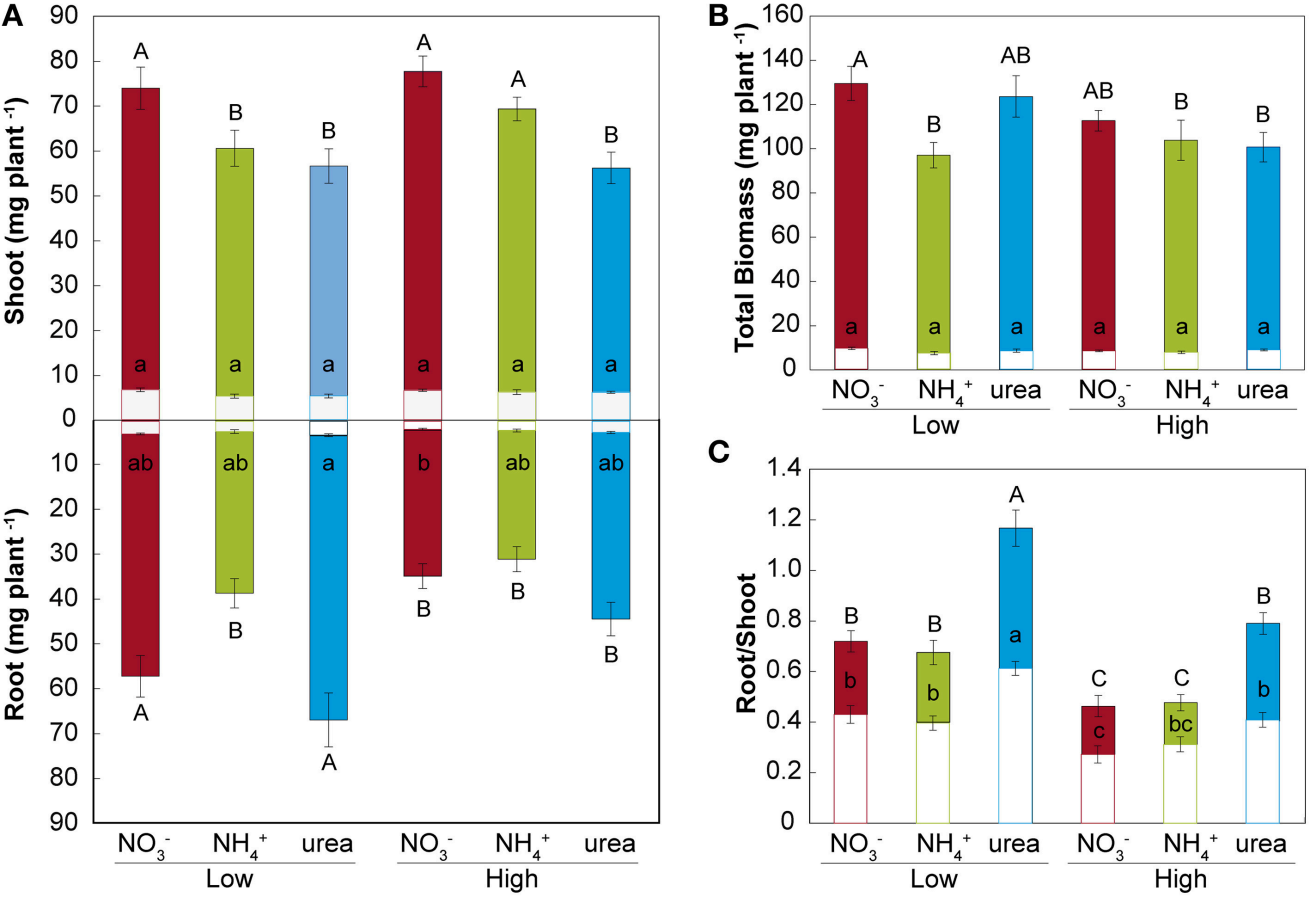
**Effects of NO${}_{3}^{-}$, NH${}_{4}^{+}$, and urea on biomass**. Distribution of the plant biomass of roots and shoots **(A)**, total biomass **(B)**, and the root/shoot ratio **(C)** expressed on the basis of the fresh weight (colored bars) and dry weight (white bars) (mg) per plant subjected to the different treatments (low and high doses of NO${}_{3}^{-}$, NH${}_{4}^{+}$, and urea). The values are the mean ± S.E (*n* = 15). Different letters (capital letters for fresh weight and lowercase letters for dry weight) denote statistically significant differences at α = 0.05 using the Student–Newman–Keuls test. To standardize the variances, one data point was replaced by the mean of the group for the root fresh weight, and 1 degree of freedom was subtracted from the residual (Winer et al., 1991).

**Figure 3:**
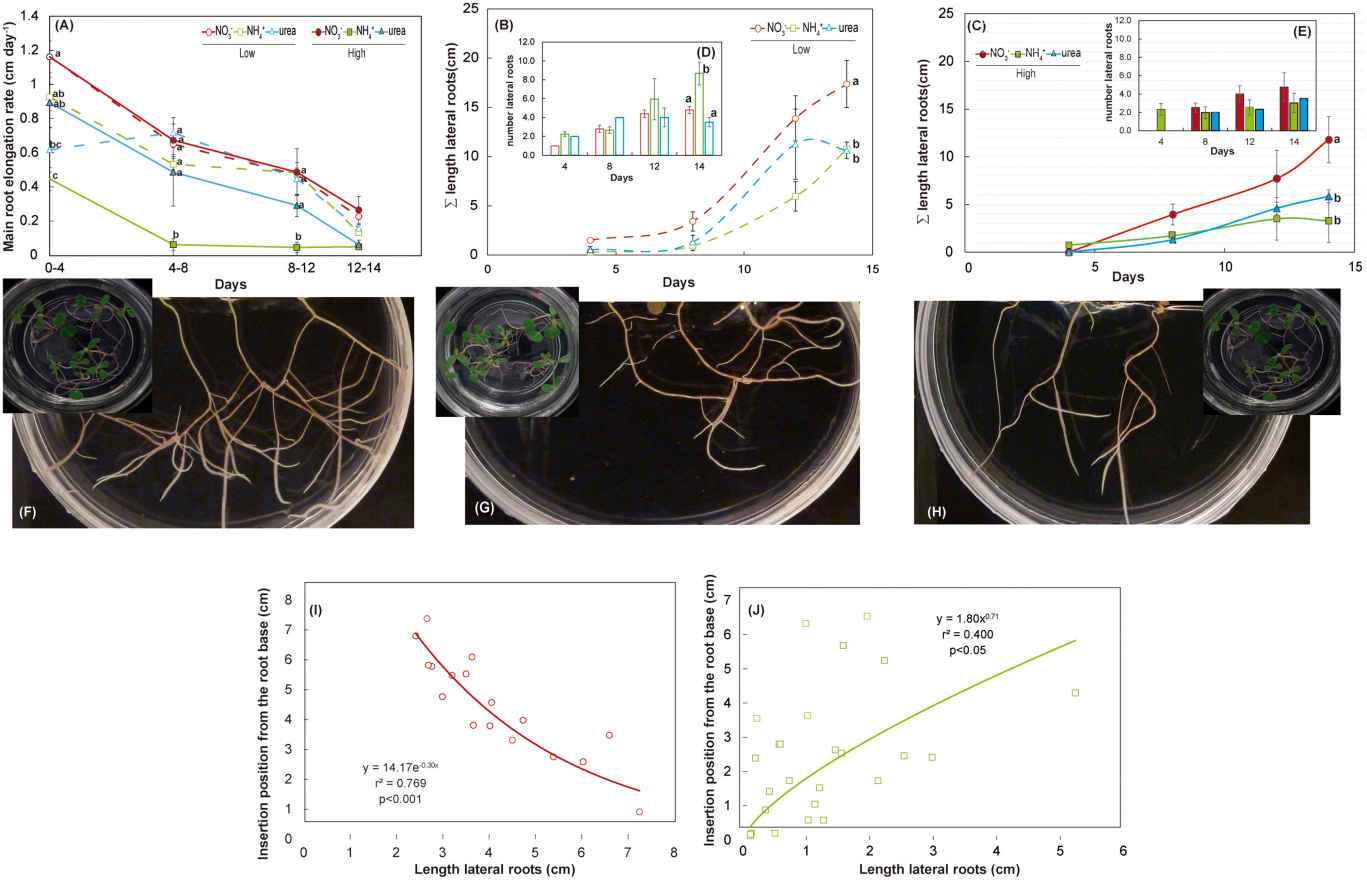
**Effects of NO${}_{3}^{-}$, NH${}_{4}^{+}$, and urea on the root system architecture**. Main root elongation rate **(A)** and accumulated (∑) lateral root length under a low dose **(B)** and high dose **(C)**. Inset panels show the changes in the number of lateral roots under each treatment for a low dose **(D)** and high dose **(E)**. The values are the mean of 5–3 ± S.E. An analysis of variance (ANOVA) was performed in **(A)** considering the treatment as a fixed factor. Different superscripted letters denote statistically significant differences at α = 0.05 using the Student-Newman-Keuls test. An absence of letters indicates that there were no significant differences. For the nitrate **(F)**, ammonium **(G)** and urea treatments at a low dose, a representative image from each condition at day 14 is shown **(H)** Relationship of the insertion position from the root base with the length of lateral roots for a low dose of NO${}_{3}^{-}$ **(I)** and a low dose of NH${}_{4}^{+}$ **(J)**. More information regarding the phenotypic analysis is provided in Supplementary Table S2.

**Figure 4:**
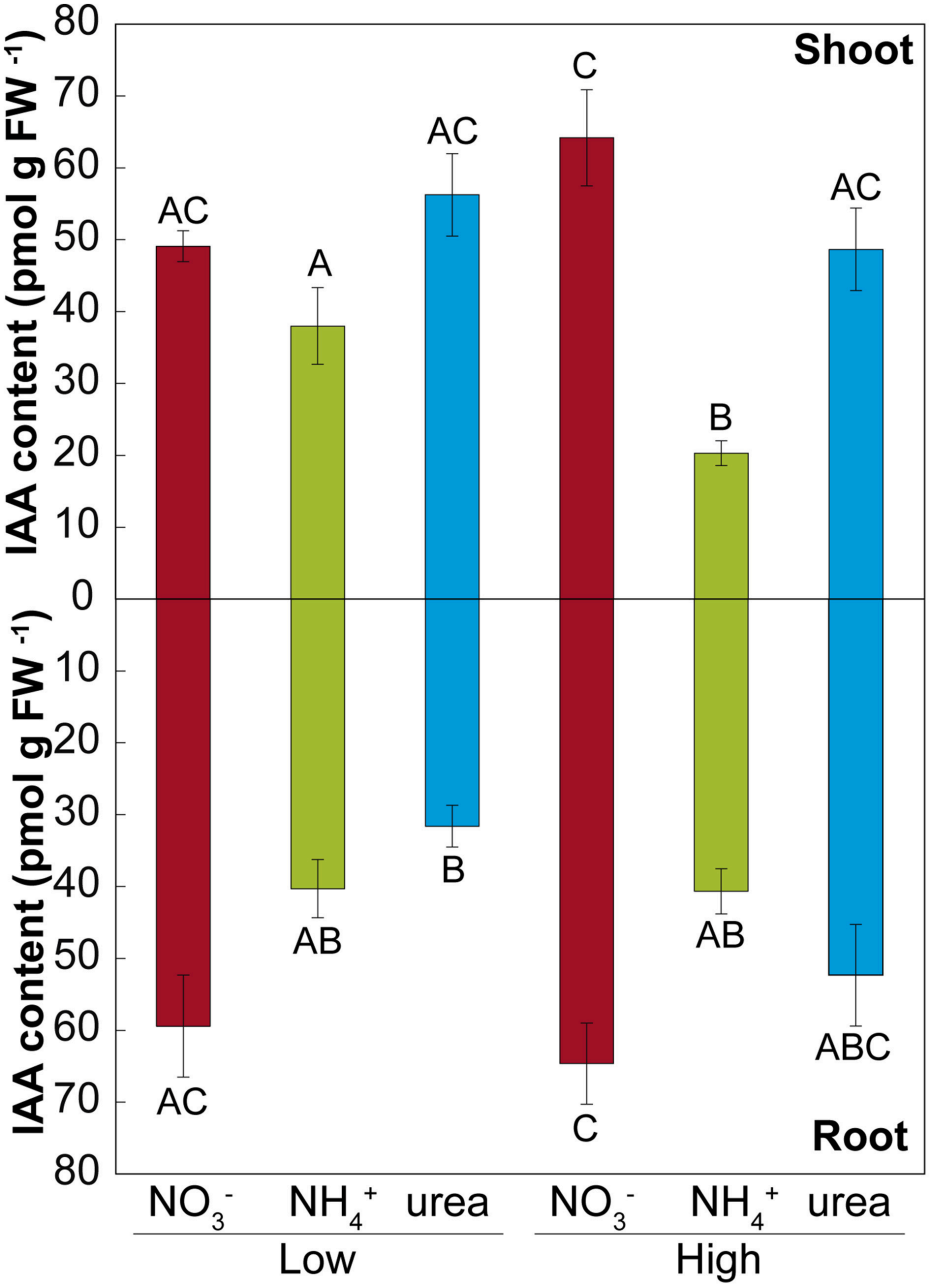
**Effects of NO${}_{3}^{-}$, NH${}_{4}^{+}$, and urea on the IAA concentration**. High and a low doses of NO${}_{3}^{-}$, NH${}_{4}^{+}$, and urea altered the IAA concentration (pmoles g^−1^ fresh weight) in *M. truncatula* seedlings. The values are the mean ± S.E (*n* = 3). Different capital letters denote statistically significant differences at α = 0.05 after the Student–Newman–Keuls test.

Treatment with either NH${}_{4}^{+}$ or urea at a low dose caused a significant reduction of the fresh weight of the shoots of *M. truncatula* seedlings compared with NO${}_{3}^{-}$ treatment (Figure 2A). At a high dose, only the plants fed with urea showed reduced shoot growth, which was related to the differential root/shoot biomass ratio, with greater investment in the roots being observed under this type of nutrition (Figure 2C). Interestingly, NH${}_{4}^{+}$ had a negative effect on fresh weight at a low dose, but not at high dose, where a different root/shoot ratio may influence changes in fresh weight. Root growth was only affected under NH${}_{4}^{+}$ nutrition at a low N concentration. The differences were not significant for dry matter in either the roots or the shoots (Figure 2B). In terms of the total biomass (Figure 2B), both low and high doses of NH${}_{4}^{+}$ decreased the growth per plant, which was also reduced under high-dose urea-fed plants. Significant changes in the architecture of the roots occurred during development (Figure 3 and Supplementary Table S2). We confirmed that there was a significant change in the main root elongation rate in each of the treatments, with a delay in growth being observed during the first 4 days in plants grown under a high dose of NH${}_{4}^{+}$ (Figure 3A). Interestingly, in the plants fed with urea at a low dose, enhancement of the elongation of the main root was observed after the first 4 days of growth. All treatments reduced the main root elongation rate after the 8th day of growth. The differences in lateral root growth and elongation were also evident and significant at the end of the experiment. In both the NH${}_{4}^{+}$- and urea-fed plants, the accumulated lateral root growth was lower than in the NO${}_{3}^{-}$-fed plants (Figures 3B,C). This differential accumulated lateral growth in the low-dose treatments was due to a greater number of lateral roots growing more slowly in NH${}_{4}^{+}$-treated roots and to the presence of fewer lateral roots in urea-fed roots (Figure 3D). No significant differences in the number of lateral roots were found for the high-dose treatments (Figure 3E). Moreover, a remarkable relationship was observed regarding the length of the lateral roots and the position of insertion from the root base, with opposite patterns being recorded in the NH${}_{4}^{+}$- and NO${}_{3}^{-}$-grown plants, as illustrated in Figures 3I,J. No significant relationship was detected in the urea-fed plants, probably due to the low number of lateral roots (data not shown; *p* = 0.075). These differences in plant growth in relation to the type of nutrition might reflect disparities in N uptake and/or assimilation, but they may also be related to differential hormone signaling. Accordingly, the free IAA content was analyzed. The NH${}_{4}^{+}$ (low and high doses)-fed plants showed a decrease in the IAA content in the roots compared with the NO${}_{3}^{-}$-grown plants (Figure 4). The decrease in the IAA content was even more pronounced and significant when analyzed based on dry weight (data not shown). However, the most remarkable decrease was observed in the shoots under 25 mM NH${}_{4}^{+}$. In contrast, urea did not alter IAA contents at the shoot level but did provoke a decrease in IAA at the root level in the low-dose treatment compared with NO${}_{3}^{-}$ treatment. Indeed, the decrease was remarkably higher in the presence of urea at a low dose than was observed for NH${}_{4}^{+}$.

### Stress indicators

**Figure 5:**
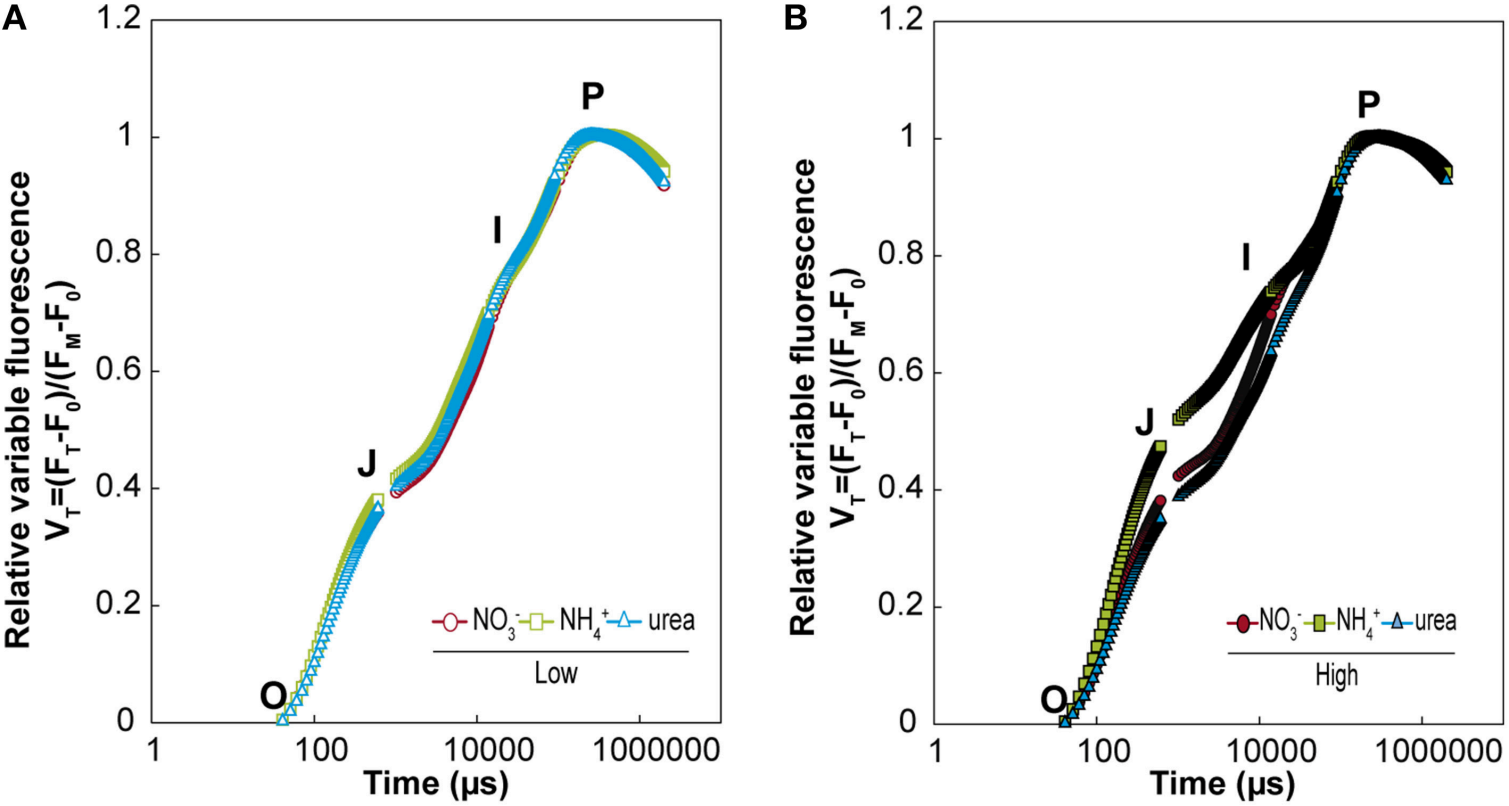
**Effects of NO${}_{3}^{-}$, NH${}_{4}^{+}$, and urea on fast chlorophyll *a* fluorescence transients (OJIP) from dark-adapted leaves of *M. truncatula* plants grown under the different treatments at low (A) and high doses (B)**. Each transient is plotted on a logarithmic time scale from 40 μs and 1 s and expressed as the relative variable fluorescence (Vt) after double normalization of Fo and Fm. The values are the means (*n* = 3–4).

Photosystem II is considered to play a key role in the response of photosynthesis to environmental stress (as may be the case for NH${}_{4}^{+}$ or ureic nutrition), and OJIP monitoring is a method for investigating the events occurring in photosystem II. This technique allows *in vivo* evaluation of plant performance in terms of biophysical parameters quantifying photosynthetic energy conservation (Strasser et al., 2000, 2004). The induction of relative variable fluorescence (Figure 5) and the parameters derived from this curve (Table 1) showed no remarkable differences between the treatments at low doses. However, there was a clear effect of a high dose of N nutrition, as indicated by the relative amplitude of the J–I phase (1–10 ms) plotted on a logarithmic time scale for NH${}_{4}^{+}$ compared with the NO${}_{3}^{-}$ and urea treatments (Figure 5). Much more pronounced differences between treatments were revealed by the parameters derived after further analyses of the curves of fluorescence according to the OJIP test (see the Material and methods and Supplementary Table S1). Indeed, the relative variable Chl fluorescence at 2 ms (V_j_) was significantly lower under high NH${}_{4}^{+}$ doses. Curiously, the maximum quantum yield of the primary photochemistry (φ_Po_) and the quantum yield for energy dissipation (φ_Do_) were significantly higher under urea at a high dose, while the efficiency with which a trapped exciton could move an electron into the electron transport chain (Ψ_o_) and the quantum yield of electron transport (φ_Eo_) were significantly lower under NH${}_{4}^{+}$ at a high dose. Regarding the specific changes per reaction center (RC), only the electron transport flux per RC (ET_*o*_/RC) was affected in high-dose NH${}_{4}^{+}$-fed plants. The parameter Pi_Abs_, which combines (i) the RC density expressed on an absorption basis, (ii) the quantum yield of the primary photochemistry, and (iii) the ability to feed electrons into the electron chain between photosystem II and I (Cascio et al., 2010), exhibited a significantly higher value under a high dose of urea, which was even higher than the value observed under NO${}_{3}^{-}$. Conversely, Pi_Abs_ showed the lowest values under NH${}_{4}^{+}$ at both doses (presenting a lower value in the high-dose treatment; Table 1). Moreover, slight differences in chlorophylls content (SPAD units) were observed, with a significant decrease being recorded in plants fed with 25 mM NH${}_{4}^{+}$. No differences in the total protein content were detected (Table 2).

**Table 1:** **Numerical values for fluorescence parameters derived from chlorophyll *a* fast florescence transients in the leaves of *M. truncatula*: (i) normalized data as V_j_ and V_i_, (ii) quantum yields and flux ratios as φ_Po_, Ψ_*o*_, φ_Eo_, (iii) performance index (Pi_*_*Abs_), and (iv) specific energy fluxes per Q${}_{\text{A}}^{-}$ reducing photosystem II centers as ABS/RC, TR_0_/RC, ET_0_/RC, and DI_0_/RC**.

**Parameter**		**Low N supply**			**High N supply**		
		**Nitrate**	**Ammonium**	**Urea**	**Nitrate**	**Ammonium**	**Urea**
Normalized data	V_*j*_	0.42±0.02^a^	0.45±0.01^a^	0.44±0.02^a^	0.45±0.03^a^	0.56±0.04^b^	0.42±0.02^a^
	V_i_	0.78±0.03	0.78±0.02	0.79±0.01	0.79±0.01	0.78±0.02	0.74±0.00
Quantum yields and flux ratios	φ_Po_	0.74±0.00^a^	0.75±0.00^ab^	0.76±0.01^ab^	0.76±0.00^ab^	0.74±0.00^a^	0.77±0.00^b^
	Ψ_*o*_	0.58±0.02^a^	0.55±0.01^a^	0.56±0.02^a^	0.55±0.03^a^	0.44±0.04^b^	0.58±0.02^a^
	φ_Eo_	0.43±0.01^a^	0.42±0.00^a^	0.43±0.02^a^	0.42±0.02^a^	0.33±0.03^b^	0.44±0.02^a^
	φ_Do_	0.26±0.00^a^	0.25±0.00^ab^	0.24±0.01^ab^	0.24±0.00^ab^	0.26±0.00^a^	0.23±0.00^b^
	Pi__Abs_	1.12±00.07^ab^	1.06±0.04^ab^	1.22±0.15^ab^	1.19±0.14^ab^	0.67±0.12^a^	1.39±0.16^b^
Specific energy fluxes	ABS/RC	3.58±0.04	3.54±0.12	3.39±0.08	3.32±0.03	3.54±0.17	3.29±0.07
	TR_o_/RC	2.67±0.04	2.66±0.07	2.57±0.04	2.53±0.08	2.63±0.11	2.52±0.04
	ET_o_/RC	1.54±0.03^a^	1.47±0.06^a^	1.44±0.03^a^	1.39±0.0^a^	1.17±0.14^b^	1.46±0.04^a^
	DI_o_/RC	0.92±0.01	0.88±0.05	0.82±0.04	0.79±0.01	0.92±0.06	0.77±0.03

*Definitions and formulae are given in the Materials and Methods and in Supplementry Table S1. The values are the means ± S.E. from independent measurements (N = 3–4). An analysis of variance (ANOVA) was performed, considering the treatments as fixed factors. Different superscripted letters denote statistically significant differences at α = 0.05 using the Student-Newman-Keuls test. An absence of letters indicates that there were no significant differences*.

**Table 2:** **Chlorophylls by SPAD units, ascorbate (ASC), and glutathione+homoglutathione (GSH+ hGSH) redox status and protein content in the tissues (shoots and roots) of *M. truncatula* plants grown using NO${}_{3}^{-}$, NH${}_{4}^{+}$, and urea media as the only N source**.

	**Tissue**	***n***	**Low**			**High**		
			**Nitrate**	**Ammonium**	**Urea**	**Nitrate**	**Ammonium**	**Urea**
Chlorophylls (SPAD units)	Leaves	14	29.1±1.2^abc^	25.9±1.5^ab^	26.3±1.6^ab^	30.8±1.0^c^	25.4±1.1^a^	29.7±1.6^bc^
ASC/(DHA+ASC)	Shoot	4	0.48±0.02^a^	0.53±0.03^ab^	0.57±0.04^b^	0.62±0.02^b^	0.53±0.00^ab^	0.58±0.02^b^
	Root	4	0.60±0.00^a^	0.57±0.0^ab^	0.57±0.02^ab^	0.54±0.02^b^	0.55±0.01^ab^	0.56±0.01^ab^
(GSH+hGSH)/(GSSG+hGSSG+GSH+hGSH)	Shoot	4	1.37±014^a^	1.29±0.21^a^	1.00±0.01^ab^	1.39±0.25^a^	0.77±0.25^ab^	0.43±0.02^b^
	Root	4	0.76±0.14^a^	0.95±0.21^a^	0.58±0.06^a^	0.73±0.07^a^	0.83±0.28^a^	0.54±0.06^a^
Protein content (mg/g^−1^ dry weight)	Shoot	8	10.4±2.0	9.6±1.6	8.2±1.7	12.5±2.0	12.5±1.8	11.4±1.8
	Root	5	8.6±1.3	8.4±1.1	8.1±1.8	11.1±1.3	10.1±1.1	8.7±1.1

*The values represent the mean ± S.E. (n = 4–14). Different superscripted letters denote statistically significant differences at α = 0.05 using the Student-Newman-Keuls test. An absence of letters indicates that there were no significantly differences*.

**Figure 6:**
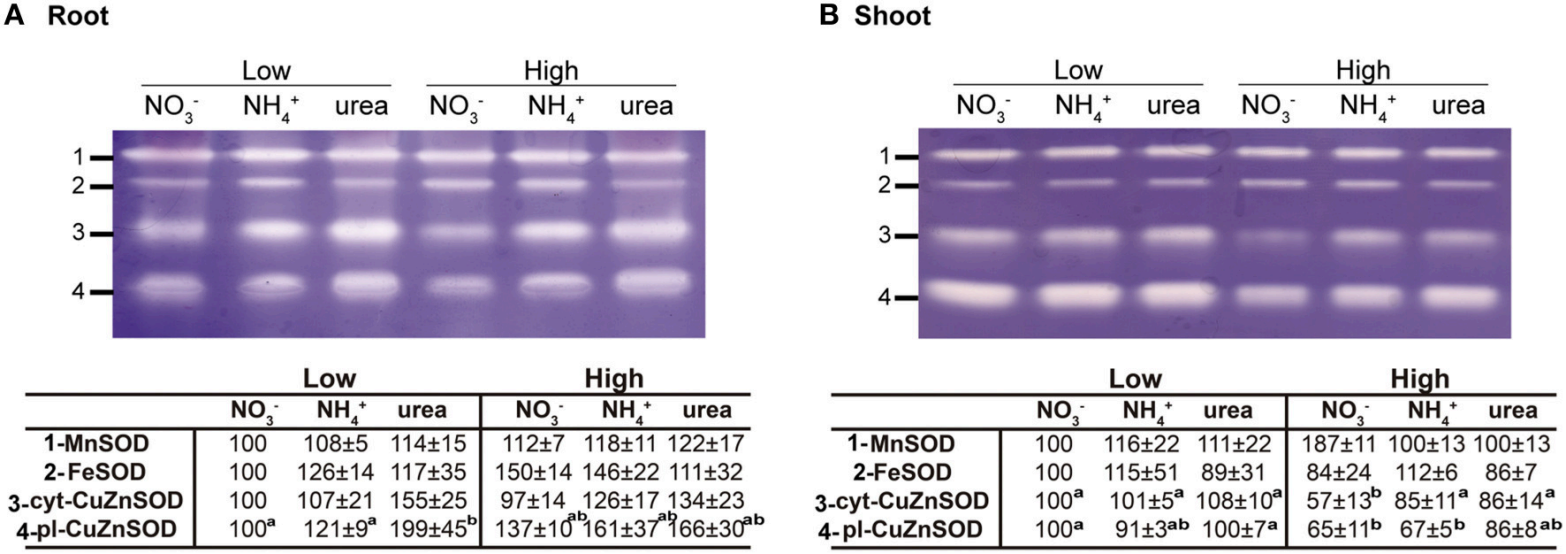
**Effect of NO${}_{3}^{-}$, NH${}_{4}^{+}$, and urea on superoxide dismutase activity in the roots (A) and leaves (B) of 2-weeks old *Medicago truncatula* plants grown under axenic conditions with NO${}_{3}^{-}$, NH${}_{4}^{+}$, or urea as the exclusive source of N**. The gels are representative examples of three replicate experiments. The densitometry results for the isoenzyme bands are expressed in the lower panel as percentages relative to the corresponding control reference bands (1 mM NO${}_{3}^{-}$). All lanes of the 15% native-PAGE gel were loaded with 30 μg of protein. The values are the means ± S.E. (*n* = 3). Different letters denote statistically significant differences at α = 0.05 using the Student–Newman–Keuls test. To standardize the variances, one data point was replaced with the mean for the group for shoot cyt-CuZnSOD and root pl-CuZnSOD, and consequently, 1 degree of freedom was subtracted from the residual in each case (Winer et al., 1991). An absence of letters indicates that there were no significant differences.

Regarding antioxidants, there were no significant changes in the size and general oxidation state of the antioxidant pools in the roots. On the contrary, the NH${}_{4}^{+}$- and urea-supplied plants presented a lower reduced pool of GSH+hGSH in the shoots. On the other hand, there were detectable differences in the ASC oxidation pool in the shoots, with higher contents being recorded in urea-grown seedlings (Table 2). Regarding SOD activity (Figure 6), four isozymes were found in both the leaves and roots (MnSOD, FeSOD, cytosolic-CuZnSOD, and plastidial-CuZnSOD). However, the response was different between the two tissues. In the roots (Figure 6A), no or very slight differences were observed for the isoenzyme MnSOD, indicating that a special level of protection for mitochondria-associated superoxide production was not required under any of the tested conditions. On the other hand, cyt-CuZnSOD was somewhat affected by the type of nutrition, increasing under low and high urea, but showing no significant ncreases under NH${}_{4}^{+}$ treatment. Increases (although not significant) could also be observed for FeSOD in plants grown under high N doses (NO${}_{3}^{-}$ and NH${}_{4}^{+}$). Unlike FeSODs from determinate nodule-forming legume plants, such as soybean, cowpea, or *Lotus japonicum*, where FeSOD members occur in the plastids or cytosol, the FeSODs of the *Medicago* family are plastidial proteins (Moran et al., 2003; Asensio et al., 2012). Additionally, plastid-associated CuZnSOD was significantly induced in plants fed with high NH${}_{4}^{+}$ and low or high urea. On the whole, it appears that plastidial enzymes are more affected, and ureic nutrition appears to impact a greater number of SOD isoenzymes. Conversely, no important changes were observed in the shoots (Figure 6B), with the exception of a decrease of cyt-CuZnSOD activity under high NO${}_{3}^{-}$ treatment. Overall, these results suggest that small adjustments in the antioxidant pool may prevent major perturbations in redox ratios under differential N nutrition.

**Figure 7:**
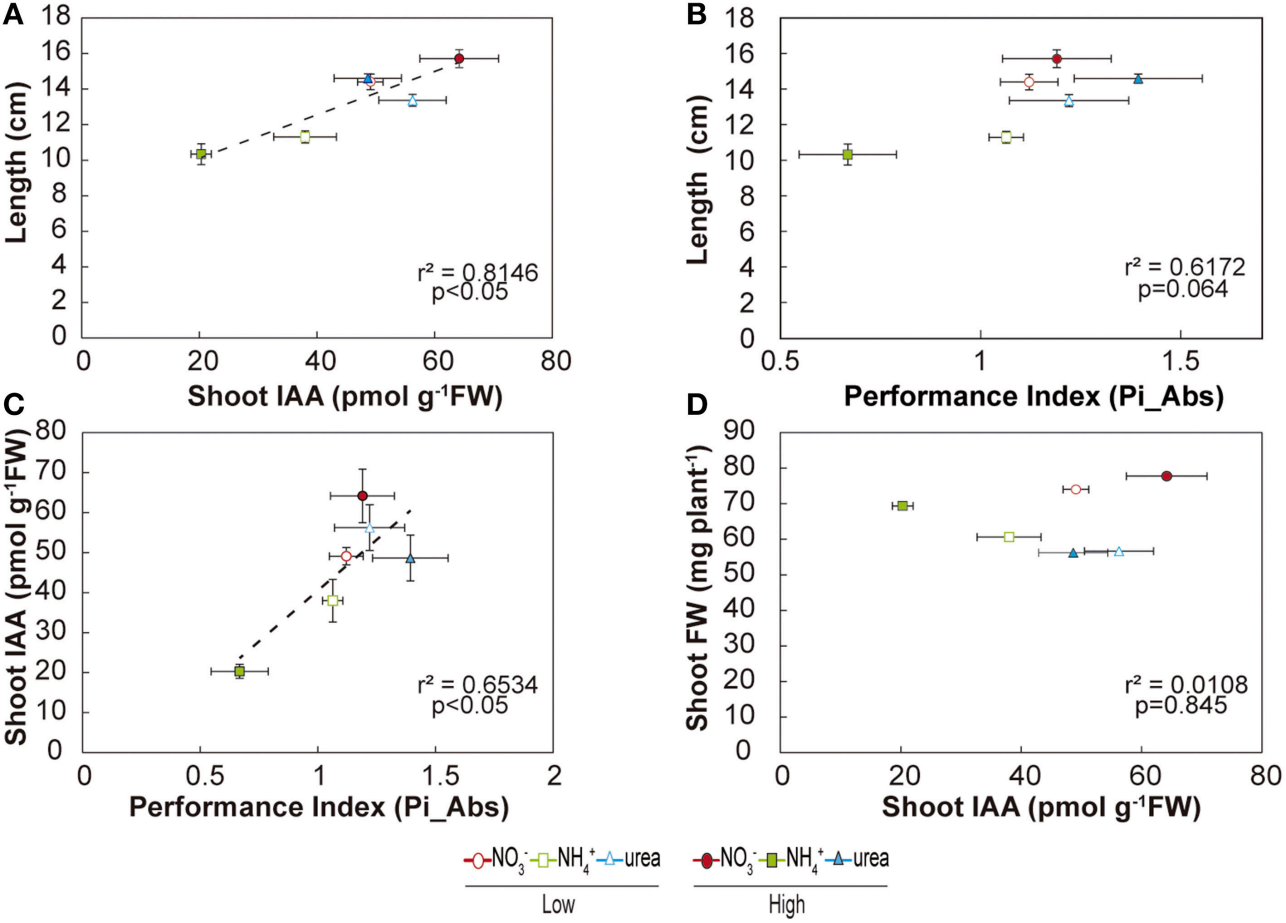
**Relationships under the effects of NO${}_{3}^{-}$, NH${}_{4}^{+}$, and urea**. Relationship of the total plant length with the shoot IAA content **(A)** and performance index **(B)**. Relationship of the shoot IAA content with the performance index **(C)** and with shoot fresh weight **(D)**. Points shown are the means (*n* = 4) ± S.E. for the plants.

Our study also demonstrated interesting relationships among the studied parameters (Figure 7). Pi_Abs_ (a parameter obtained from the induction of chlorophyll *a*) showed a significant positive relationship with the shoot IAA content (*r*^2^ = 0.653; *p* < 0.05; Figure 7C). Moreover, the shoot IAA content exhibited a significant positive relationship with the total length of the plant (*r*^2^ = 0.815; *p* < 0.05), as did the total IAA content with the total length of the plant (data not shown; *r*^2^ = 0.778; *p* < 0.05). There was also a tendency for a greater length to be correlated with a higher performance index, although not significantly (*r*^2^ = 0.617; Figure 7B). Interestingly, total biomass on a fresh basis was not correlated with the IAA content (Figure 7D), nor was the root IAA content correlated with root length (data not shown). Taken together, these findings suggested that NO${}_{3}^{-}$- and urea-fed seedlings showed higher performance indices than NH${}_{4}^{+}$-fed plants, which was clearly correlated with higher IAA contents, potentially resulting in better length growth for these plants.

## Discussion

### The importance of pH control

Growing crop plants using NH${}_{4}^{+}$ or urea as the only N source under axenic conditions is a poorly examined method. Few studies are performed under these conditions, especially in plants grown with urea (Gerendás et al., 1998; Wang et al., 2013; Yang et al., 2015), and it is therefore a challenging task. Appropriate control of the pH in the medium after the sterilization process is important to achieve the correct growth of plants and appropriate evaluation of urea or NH${}_{4}^{+}$ nutrition treatments because the autoclaving process may change the pH of the solution when certain nutrients are autoclaved together (see the Materials and Methods). Moreover, it is widely known that the pH of the medium may have an effect on nutrient assimilation during the development of seedlings. Our system standardized the control of pH during the preparation of the media and the development of the seedlings using the routine color indicator methyl red (Figure 1). Our results showed that the pH did not decrease greatly under all tested conditions, except at a very high dose of NH${}_{4}^{+}$, where the pH could drop to 4.5. Under this condition, the plants showed a maximum quantum yield of primary photochemistry similar to the rest of the treatments (φ_Po_; Table 1). In contrast, in non-buffered full-strength solutions containing NH${}_{4}^{+}$, pH values lower than 4.0 can occur, which renders the solution unsuitable for healthy growth (Zaccheo et al., 2006).

### How are the shoots and roots affected by NH${}_{4}^{+}$ and urea nutrition?

One of the most dramatic plant adaptations for ensuring adequate N acquisition is the modulation of the RSA (Forde and Lorenzo, 2001; Hodge, 2004; Smith and De Smet, 2012; Forde, 2014). A detailed description of root development under NH${}_{4}^{+}$ nutrition has not yet been provided, and only a few studies have described the changes in the RSA under urea nutrition (Yang et al., 2015; Zanin et al., 2015). Our results obtained in both NH${}_{4}^{+}$- and urea-fed plants at both tested doses (Figures 1A, 3A–C) agree with the phenotype described under NH${}_{4}^{+}$ stress in the majority of plants (Britto and Kronzucker, 2002). These changes imply the existence of shortened roots, with a significant reduction of the primary root, which is explained by the inhibition of cell elongation (Li et al., 2010) and the suppression of lateral root elongation (Li et al., 2010, 2011; Rogato et al., 2010). Inhibition of lateral root length was also observed under a high NO${}_{3}^{-}$ supply (Figure 3C; Walch-Liu et al., 2006). Interestingly, we found a negative correlation for NO${}_{3}^{-}$-grown roots (Figure 3I) and a positive relationship for NH${}_{4}^{+}$-grown roots (Figure 3J) when relating the position of insertion from the base of the lateral roots to their lengths. The absence of a relationship in urea-fed plants was probably due to the small number of lateral roots in these plants (Figure 3B), as reported for urea-grown *Arabidopsis* plants (Yang et al., 2015). Conversely, Zanin et al. (2015) described a significant increase in the total density and area of urea-fed maize roots.

Our results did not show a decrease in dry weight in any of the treatments, probably due to the low biomass originating from axenic cultures. Other species described as tolerant to NH${}_{4}^{+}$ nutrition, such as *M. truncatula*, have not been found to show any decrease in dry matter contents when grown with NH${}_{4}^{+}$ as the only N source (Domínguez-Valdivia et al., 2008; Cruz et al., 2011). Although there were no significant changes in biomass, the root/shoot ratio was significantly altered under different N sources, as previously demonstrated for high and low N doses (Ariz et al., 2011a). The urea-treated plants showed significant growth reduction on a fresh weight basis, compared with the plants grown under NO${}_{3}^{-}$ nutrition, but to a lesser extent than the NH${}_{4}^{+}$-grown seedlings, as described for a number of plant species (Houdusse et al., 2005; Mérigout et al., 2008; Garnica et al., 2010; Yang et al., 2015). Indeed, various authors have reported that plants fed with urea suffer from N deficiency due to an extremely low urea uptake rate (Arkoun et al., 2012; Wang et al., 2013). However, the recent description of efficient urea transporters (Kojima et al., 2007; Wang et al., 2008; Zanin et al., 2014; Liu et al., 2015) confirms the possibility that plants may use urea the sole nitrogen source, with the compound being assimilated and translocated by plants (Mérigout et al., 2008). The unchanged total biomass of urea- and NO${}_{3}^{-}$-fed plants (Figure 2B) showed that urea is taken up by the roots without hydrolysis in our experimental system, as the axenic conditions prevented any microbial-based conversion of the supplied N forms during the course of the experiments. Furthermore, no urea degradation was found to occur in the nutrient media, as no NH${}_{4}^{+}$ was detected through ion chromatography in samples of the agar media collected randomly at the end of plant growth (data not shown). Finally, the similar protein contents of the root tissues after exposure to the different N sources indicated efficient assimilation of urea and NH${}_{4}^{+}$ in our plants (Table 2). Accordingly, Cao et al. (2010) reported that urea-grown plants were subject to a more inefficient N distribution than to inefficient urea uptake, and it was subsequently reported that plants are able to use urea (Zanin et al., 2015).

### How is the IAA pool affected by NH${}_{4}^{+}$ and urea nutrition?

Phenotypic changes, beyond simple morphological transformation, may imply more profound regulation regarding hormone-related signals (Guo et al., 2009; Bartoli et al., 2013). Accordingly, auxin levels have been reported to regulate the elongation of the main and lateral roots during development (Péret et al., 2009; Li et al., 2011). In the present study, we did not observe inhibition of IAA levels in the roots under a high NO${}_{3}^{-}$ supply (Figure 4), as has been described in other studies (Tian et al., 2008; Tamura et al., 2010), which may likely explain why the primary length was not inhibited under a high NO${}_{3}^{-}$ supply in our experiments (Figure 3A). Conversely, in NH${}_{4}^{+}$-fed-plants, a suppression of root IAA contents has been described (Kudoyarova et al., 1997; Li et al., 2011), as observed in the present work (Figure 3C). In urea-supplied plants, transcriptomic data have also shown a marked phytohormonal imbalance, although auxin-regulated gene categories were not affected (Yang et al., 2015). This may be explained by the absence of NO${}_{3}^{-}$, in urea- and NH${}_{4}^{+}$-grown seedlings. Nitrate is essential for the uptake of IAA into root cells and therefore for auxin signaling (Krouk et al., 2010). Thus, in treatments with a complete absence of NO${}_{3}^{-}$, the length of lateral roots may be altered due to the absence of signaling related to the NO${}_{3}^{-}$-auxin interplay in the roots (Krouk et al., 2010). On the other hand, the correlation found between total length and the shoot IAA pool in *M. truncatula* (Figure 7A) suggests that exogenous IAA could complement the NH${}_{4}^{+}$ and urea phenotype. Accordingly, IAA was added to the growth medium at 20 μM. However, it did not promote root development (data not shown). Barth et al. (2010) and Yang et al. (2015) also concluded that plants grown with exogenous IAA did not show any symptoms of growth recovery from NH${}_{4}^{+}$ toxicity or urea nutrition, respectively. The shoots of NO${}_{3}^{-}$-fed plants presented higher IAA levels than those of urea-fed plants (at high doses), in contrast to what was observed by Mercier et al. (1997), who reported that urea-treated plants presented higher IAA levels than those grown with NO${}_{3}^{-}$. Nitrate may be not as important in the shoots as in the roots, as we observed that the IAA contents were positively correlated with plant length and the performance index, indicating differences in regulation and effects for both the roots and shoots. Taking into account that the suppression of auxin signaling is considered to enhance stress tolerance (biotic and abiotic) (Park et al., 2007), the decline of IAA contents observed under urea and NH${}_{4}^{+}$ nutrition (more pronounced) suggests that both treatments may modify auxin contents, suggesting a trade-off mechanism for enhancing tolerance toward these conditions.

### Do urea and NH${}_{4}^{+}$ nutrition induce stress in *M. truncatula*?

Although the toxic effect of NH${}_{4}^{+}$ at the whole-plant level is widely known (Britto and Kronzucker, 2002; Bittsánszky et al., 2015), the toxic effect of urea is still a matter of debate. The effects of NH${}_{4}^{+}$ on the photosynthetic machinery have been described in several species, such as spinach (Lasa et al., 2002), maize (Foyer et al., 1994), and *Synechocystis* (Drath et al., 2008). The similar maximum quantum yield of the primary photochemistry observed in all of the treatments (φ_Po_) revealed that the photosynthetic apparatus of *M. truncatula* was not photoinhibited under different types of nutrition, as shown in other studies (Podgórska et al., 2013; Bittsánszky et al., 2015). However, a detailed analysis of the kinetic transients of chlorophyll *a* fluorescence indicated that NH${}_{4}^{+}$ at a high dose was associated as the most sensitive phenotype, due to the lower value of Pi_Abs_ (Figure 5 and Table 1). Paradoxically, urea, which has been reported to share similar assimilation pathways in *Arabidopsis* (Mérigout et al., 2008), had the opposite effect. Not only did urea-treated *M. truncatula* plants maintain their photosynthetic capacity, but the biophysical parameters of the photosynthetic apparatus also indicated that urea-grown plants displayed a dose-dependent improvement in energy conservation from absorbed photons to reduction, which was even better than that observed in NO${}_{3}^{-}$-grown plants. Urea applied at the leaf level has been reported to increase leaf CO_2_ assimilation in N-deficient plants (Del Amor and Cuadra-Crespo, 2011). However, transcriptomic data indicated down-regulation of genes involved in photosynthesis (Yang et al., 2015). The investigation of whether oxidative stress is involved in NH${}_{4}^{+}$ tolerance/toxicity has led to contradictory conclusions. Ammonium does not give rise to oxidative stress in pea and spinach plants (Domínguez-Valdivia et al., 2008). However, it does generate stress in aquatic plants (Nimptsch and Pflugmacher, 2007), tobacco (Skopelitis et al., 2006), and *Arabidopsis* (Patterson et al., 2010). Most plants exhibit more oxidized states for both antioxidants when NH${}_{4}^{+}$ is supplied (Podgórska and Szal, 2015). However, we did not find clear differences in the redox status of ASC and GSH+hGSG (Table 2), and mitochondria-associated superoxide production was not specifically increased under any of the conditions, in contrast to the findings of other studies conducted in *A. thaliana*, in which mitochondrial Mn-dependent SOD has been shown to be particularly upregulated when NH${}_{4}^{+}$ is supplied (Podgórska et al., 2013; Podgórska and Szal, 2015). Our results suggested that plastidial enzymes were more affected by both NH${}_{4}^{+}$ and urea, while urea nutrition impacted the greatest number of SOD isoenzymes, which suggests that production of ROS occurs in this situation. This observation is consistent with a transcriptomic analysis performed in urea-fed *Arabidopsis* plants, in which FeSOD genes were found to be overexpressed (Mérigout et al., 2008). In our model, the legume *M. truncatula* did not appear to suffer from NH${}_{4}^{+}$ toxicity, as the leaves did not show any chlorotic symptoms, which are the main markers of NH${}_{4}^{+}$ toxicity (Britto and Kronzucker, 2002). This finding agrees with the fact that legume plants are relatively tolerant to NH${}_{4}^{+}$, as demonstrated by the growth ratio between NO${}_{3}^{-}$ and NH${}_{4}^{+}$ conditions (Ariz et al., 2011a). Overall, NH${}_{4}^{+}$ treatment leads to “mild” perturbation under this system, with differential effects on the photosynthetic machinery.

## Conclusion

Taken together, our results showed that low N doses from different sources had no remarkable effects on *M. truncatula* plant performance. The exception was the modification of RSA, resulting in changes in both lateral root development and the relationship of the insertion position from the root base, which were clearly affected by both NH${}_{4}^{+}$ and urea treatments and demonstrated great plasticity and modulation of *M. truncatula* roots. Conversely, our system revealed that *M. truncatula* grown under NH${}_{4}^{+}$ nutrition at a high dose presented a lower performance index and enhanced activity of SOD; however, we did not find clear symptoms of a reduction in the plant antioxidant status. In plants grown with urea at a high dose, we observed SOD up-regulation, but no effect on the photosynthetic apparatus was detected. Therefore, in both plant treatments, these reactive species may be more involved in the activation of the defense system, rather than the induction of cellular damage. Legumes have been demonstrated to be more tolerant to NH${}_{4}^{+}$ than other crops (Ariz et al., 2011a) and are probably also more tolerant to urea. Our results indicate that rather than being characterized by a physiological disorder, urea-fed plants experienced a modest alteration of some cellular parameters (redox state and growth). Taken together, our findings indicate that NH${}_{4}^{+}$ at high dose, but not urea, represents a more stressful condition for *M. truncatula* in terms of biophysical parameters, with modification of the IAA pool being observed at the shoot level. Our results indicate that both the IAA pool and performance index are important players in the response of the plants to NH${}_{4}^{+}$ or urea as the sole source of nitrogen.

## Author contributions

RE and BR conceived and performed experiments, interpreted data, and contributed to the drafting of the manuscript. RE also supervised the whole project and wrote the manuscript. EU performed experiments and contributed to the correction of the manuscript. AZ and JG performed IAA analyses and contributed to the drafting of the manuscript and JFM conceived and supervised the whole project and gave experimental advice and contributed to the manuscript.

## Funding

This work was supported by the grants AGL2010-16167 and AGL2014-52396-from the Spanish Ministry of Economy and Competitiviness-Mineco. BR is a holder of a PhD fellowship from the Mineco. RE received a JAE-Doc-2011-046 contract from the Spanish Research Council (CSIC) of the programme ≪Junta para la Ampliación de Estudios≫ co-financed by the European Social Fund.

### Conflict of interest statement

The authors declare that the research was conducted in the absence of any commercial or financial relationships that could be construed as a potential conflict of interest.

## Abbreviations

ASC: ascorbate
IAA: indole-3-acetic acid
DHA: dehydroascorbate
GSH: glutathione
GSSG: oxidized glutathione
hGSH: homoglutathione
hGSSG: oxidized homoglutathione
jDo: quantum yield for energy dissipation
jEo: quantum yield of electron transport
jPo: quantum yield of primary photochemistry
N: nitrogen
NH${}_{4}^{+}$: ammonium
NO${}_{3}^{-}$: nitrate
OJIP: chlorophyll *a* fluorescence transients
Pi_Abs_: performance index
yo: efficiency with which a trapped exciton can move an electron into the electron transport chain
RC: reaction center
ROS: reactive oxygen species
RSA: root system architecture
SOD: superoxide dismutase.
