# Supplementary material for: Both Free Indole-3-Acetic Acid and Photosynthetic Performance are Important Players in the Response of Medicago truncatula to Urea and Ammonium Nutrition Under Axenic Conditions
Source: Front Plant Sci. 2016 Feb 16;7:140. doi: 10.3389/fpls.2016.00140 (PMC4754419; doi:10.3389/fpls.2016.00140)
Supplement: Supplementary file 1 [file Table1.docx]

**Supplementary Table S1.** Definition of the terms and formulae of the OJIP test parameters used for the analysis of chlorophyll *a* fluorescence transients shown in Figure 5 and Table 1, following the formulae of Strasser et al. (2000, 2004).

| **Data extracted from the recorded fluorescence transient OJIP** | | | | | | | | | |
| --- | --- | --- | --- | --- | --- | --- | --- | --- | --- |
| F_t_ |  | | | | | | | Fluorescence at time t after onset of actinic illumination | |
| F_o_ | | | | = F_50 µs_ | | | | Minimal fluorescence intensity at 50 µs, when all reaction centers (RCs) are open | |
| F_j_ | | | | =F_2ms_ | | | | Fluorescence value at 2 ms (J-level) | |
| F_i_ | | | | =F_30ms_ | | | | Fluorescence value at 30 ms (I-level) | |
| F_M_ | | | | =F_p_=F_1s_ | | | | Maximal fluorescence intensity, when all RCs are closed | |
| M_0_ | | | | 4(F_300µs_–F_0_)/(F_M_–F_0_) | | | | initial slope of the fluorescence transient | |
| S_M_ | | | | Area/(F_M_-F_o_) | | | | Normalized area (assumed proportional to the number of reduction and oxidation of one Q_A_^-^molecule during the fast  OJIP transient, and therefore related to the number of electron carriers per electron transport chain) | |
| **Fluorescence parameters derived from the extracted data** | | | | | | | | | |
| V_t_ | | | | | =(F_t_-F_0_)/(F_M_-F_0_) | | | | Relative variable chlorophyll (Chl) fluorescence at time t (from F_0_ to F_M_) |
| V_j_ | | | | | =(F_J_-F_O_)/(F_M_-F_0_) | | | | Relative variable Chl fluorescence at 2 ms (at the J-step) |
| V_i_ | | | | | =(F_i_-F_O_)/(F_M_-F_0_) | | | | Relative variable Chl fluorescence at 30 ms (at the I-step) |
| **Specific energy fluxes per RC**  (where TR, ABS and ET denote the trapped, the absorbed excitation energy fluxes and the electron transport rate respectively) | | | | | | | | | |
| ABS/RC | | | | (M_0_/V_J_)^.^F_M_/(F_M_–F_0_) | | | Specific flux for absorption: absorption flux per RC. Also a measure of PSII apparent antenna size | | |
| TRo/RC | | | | M_0_/V_J_ | | | Specific flux for trapping: trapped energy flux per RC resulting in the reduction of QA to QA^–^ | | |
| ETo/RC | | | (M_0_/V_J_)(1–V_J_) | | | | Specific flux for electron transport: electron transport flux per RC | | |
| DIo/RC | | | (M_0_/V_J_)(F_0_/F_V_) | | | | Specific flux for dissipation: the excitation energy dissipated, mainly as heat and less as fluorescence emission per RC | | |
| **Quantum yields or flux ratio** | | | | | | | | | |
| φ_Po_ | | F_v_/F_M_ | | | | The maximum quantum yield of primary photochemistry: represents the probability that an absorbed photon is trapped by the RC and used for primary photochemistry. | | | |
| Ψ_o_ | | 1 - V_J_ | | | | The efficiency with which a trapped exciton can move an electron into the electron transport chain further than QA. | | | |
| φ_Eo_ | | (1–(F_0_/F_M_)). Ψo | | | | The quantum yield of electron transport: represents the probability that an absorbed photon moves an electron into the electron transport chain | | | |
| φ_Do_ | | 1– φ_Po_–(F_0_/F_M_) | | | | The quantum yield for energy dissipation | | | |
| PI_Abs_ | [RC/ABS][TR_0_/(ABS-TR_0_)][ET_0_/(TR_0_-ET_0_) | | | | | | | | Performance index (potential) for energy conservation from photons absorbed by photosystem II to the reduction of intersystem electron acceptors. |
