# Supplementary material for: Both Free Indole-3-Acetic Acid and Photosynthetic Performance are Important Players in the Response of Medicago truncatula to Urea and Ammonium Nutrition Under Axenic Conditions
Source: Front Plant Sci. 2016 Feb 16;7:140. doi: 10.3389/fpls.2016.00140 (PMC4754419; doi:10.3389/fpls.2016.00140)
Supplement: Supplementary file 2 [file Table2.docx]

**Supplementary Table S2.** Phenotypic evaluation of the effects of various N treatments on the root system architecture in fifteen-day-old *M. truncatula* seedlings, grown under low and high doses of different N sources: NO_3_^-^, NH_4_^+^ and urea. The analyses of root system architecture were performed using SmartRoot software. This software converts pixel data into cm units. The data are the means ± SD based on four or five independent biological replicates. Different small letters denote statistically significant differences at α=0.05 using the Student-Newman-Keuls test.

|  | **Treatment** | **Primary root length**  **(cm)** | **Primary root surface**  **(cm^2^)** | **Lateral root**  **length**  **(cm)** | **Lateral root**  **Surface**  **(cm^2^)** |
| --- | --- | --- | --- | --- | --- |
| *Low* | **NO_3_^-^**  **NH_4_^+^** | 9.6±0.2**^a^**  6.6±1.2**^a^** | 2.2±0.1**^a^**  1.5±0.3**^a^** | 3.6±0.3**^a^**  1.2±0.2**^bd^** | 0.6±0.1**^a^**  0.2±0.0**^b^** |
|  | **Urea** | 7.5±0.2**^a^** | 1.5±0.2**^a^** | 3.0±0.9**^c^** | 0.6±0.2**^a^** |
| *High* | **NO_3_^-^** | 11.1±1.1**^a^** | 1.8±0.3**^a^** | 1.2±0.3**^bd^** | 0.1±0.0**^b^** |
|  | **NH_4_^+^** | 2.0±0.3**^b^** | 1.8±0.2**^a^** | 0.7±0.1**^d^** | 0.1±0.0**^b^** |
|  | **Urea** | 6.9±1.6**^a^** | 1.5±0.2**^a^** | 2.2±0.4**^bc^** | 0.6±0.1**^a^** |
